# Supplementary material for: Endosymbiont and gut bacterial communities of the brown-banded cockroach, Supella longipalpa
Source: PeerJ. 2024 Mar 19;12:e17095. doi: 10.7717/peerj.17095 (PMC10959106; doi:10.7717/peerj.17095)

Supplemmentary 1A

S. longipalpa **with** *Blattabacterium* and *Wolbachia* Alpha Diversity (ASV Level)

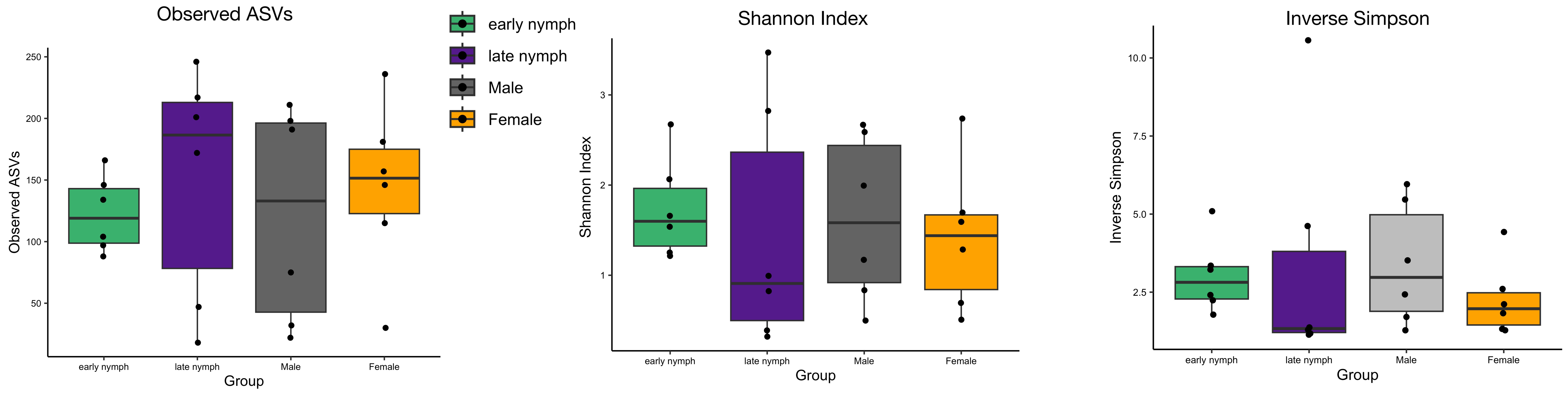

S. longipalpa **without** *Blattabacterium* and *Wolbachia* Alpha Diversity

Supplemmentary 1B

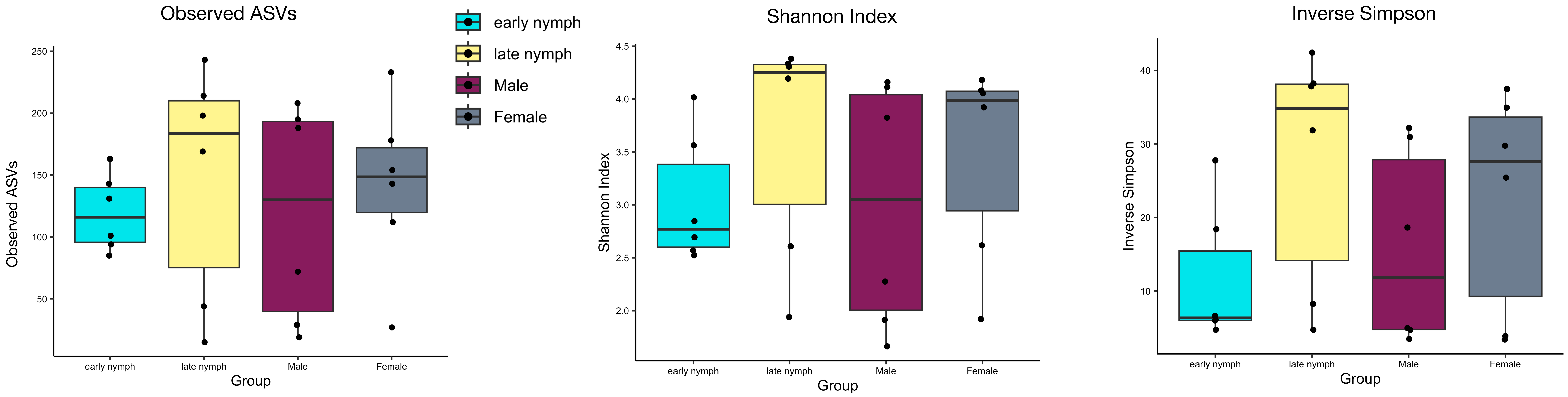

Supplementary 2A, 2B, 2C

Bray-Curtis Distances **without** endosymbionts (**genus level**)

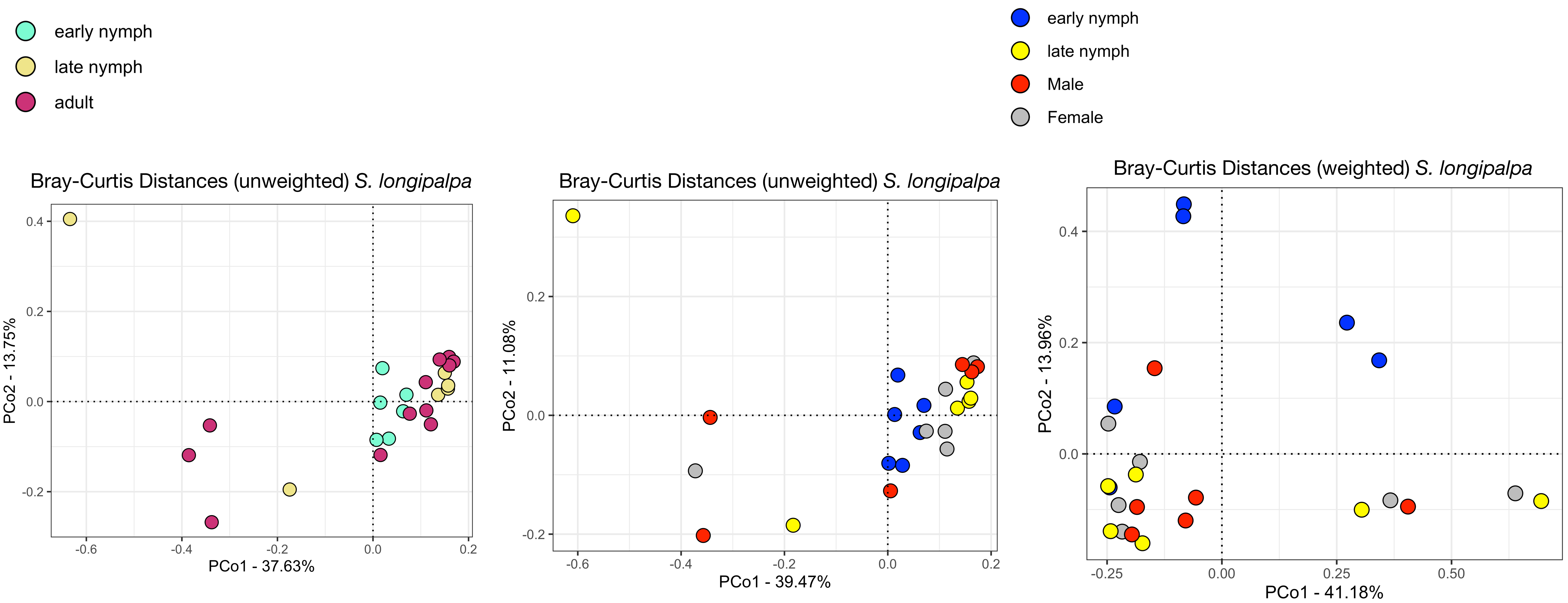

PERMANOVA: R2=0.09; F-statistic=1.05; p-value=0.35

PERMANOVA: R2=0.08; F-statistic=0.65; p-value=0.89

PERMANOVA: R2=0.14; F-statistic=1.15; p-value=0.32

Supplementary 3A, 3B, 3C

Differential Abundance Analysis of *S. longipalpa* by stages (early nymph, late nymph, adult)
(Boxplots illustrating the centered-log ratio transformation of abundances)

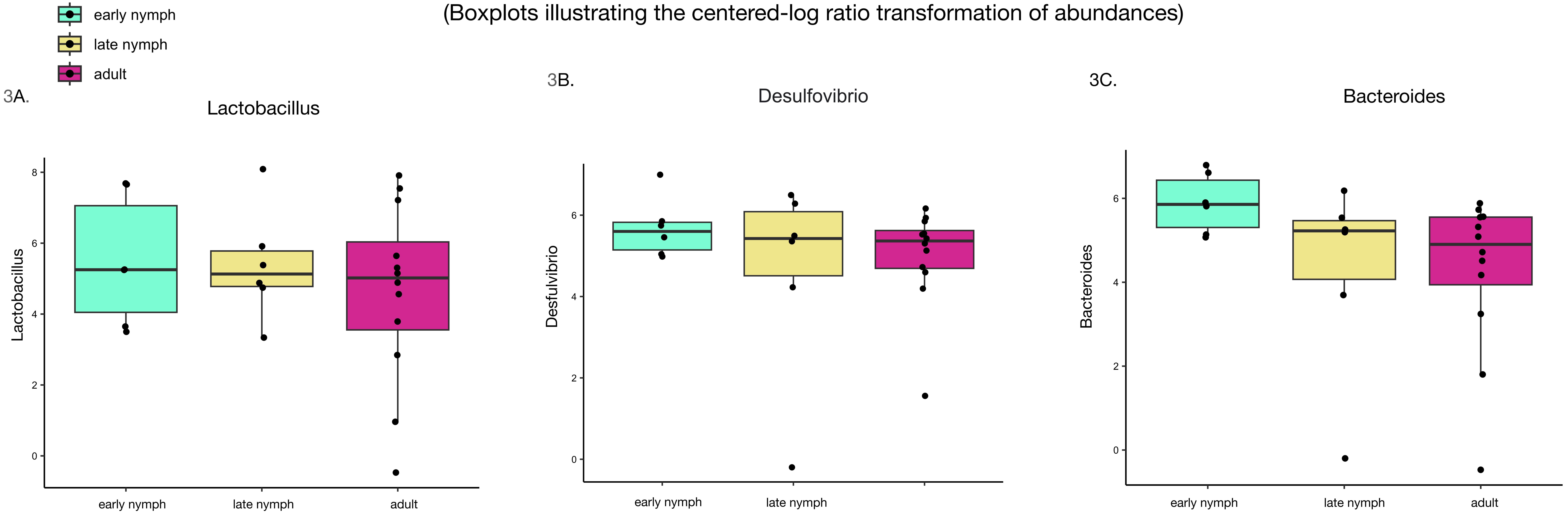

Supplementary 4A-4F

Differential Abundance Analysis of *S. longipalpa* Males and Females
(Boxplots illustrating the centered-log ratio transformation of abundances)

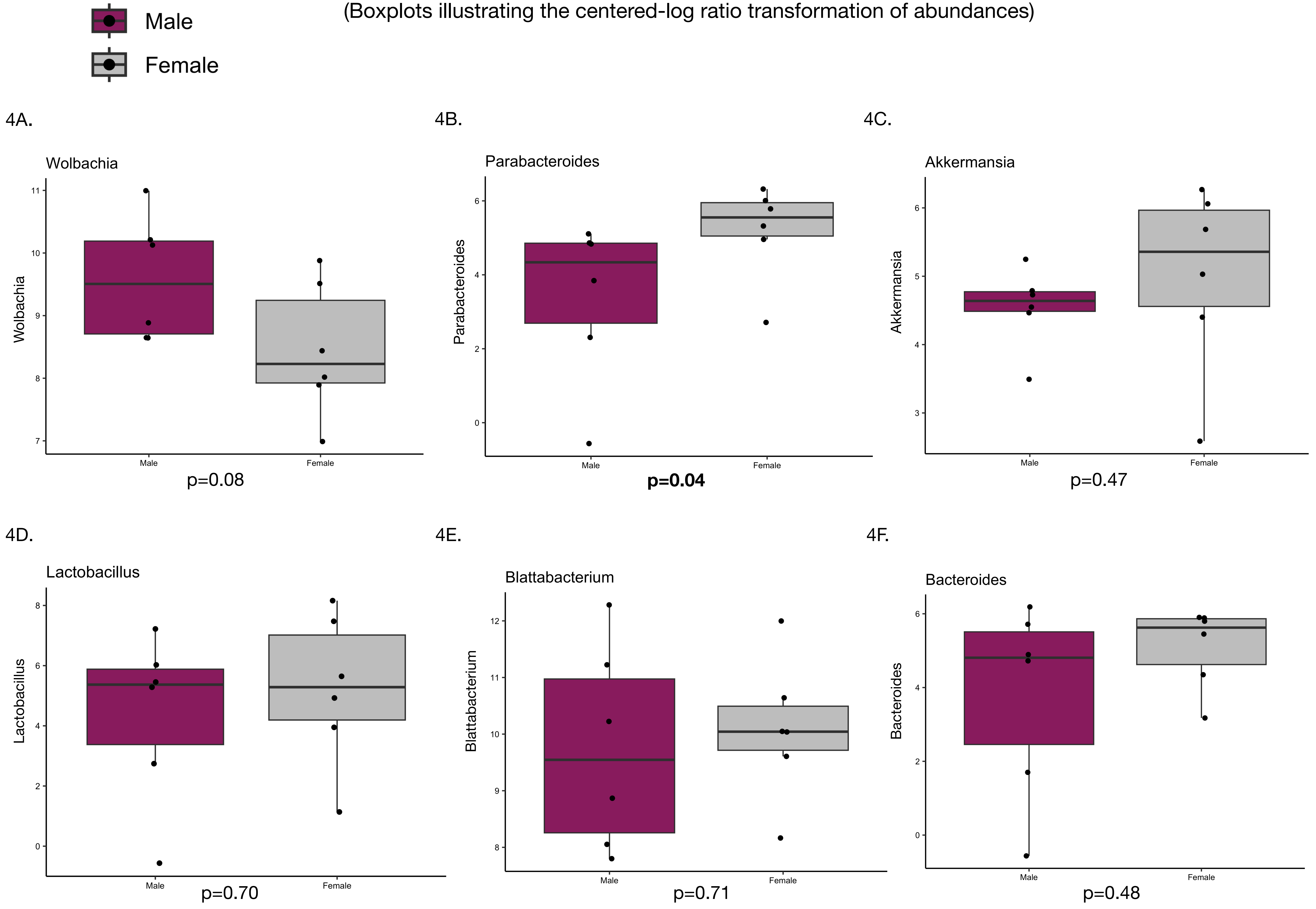

Supplement: Supplemental Information 1 [file peerj-12-17095-s001.pdf]
